# Evaluation of a prototype bioreactor based on slowly rotating drum

**DOI:** 10.1007/s10616-026-01036-1

**Published:** 2026-07-17

**Authors:** Alfredo Ambrico, Mario Trupo, Rosaria Alessandra Magarelli, Vincenzo Larocca, Maria Martino, Sergio Modenese, Sirio Vurro

**Affiliations:** 1 Department for Sustainability, Trisaia Research Center, ENEA-Italian National Agency for New Technologies, Energy and Sustainable Economic Development, Rotondella, 75026 Italy; 2 Eco-Sistemi Srl, Rovereto, 38068 Italy

**Keywords:** Monoclonal antibodies (mAbs), Chinese hamster ovary (CHO) cells, Volumetric mass transfer coefficient (k_L_a), Bioreactors, Biopharmaceuticals

## Abstract

In this study, an innovative rotating-drum bioreactor was evaluated and compared with a conventional stirred-tank system for CHO cell fed-batch culture, with particular focus on oxygen transfer, hydrodynamic conditions, and overall bioprocess performance. The novel configuration, based on a slowly rotating perforated drum, enabled efficient oxygenation at low rotational speeds, achieving a higher volumetric mass transfer coefficient (k_L_a = 0.1087 min⁻¹) despite significantly lower tip speeds. This resulted in stable dissolved oxygen (DO) levels (approximately 40%) throughout a 10-day cultivation. Under these operating conditions, the innovative bioreactor promoted enhanced cell growth, achieving a peak viable cell density (VCD) of 2.55 × 10⁷ cells mL⁻¹ and maintaining high viability (around 91.7% on day 10). Monoclonal antibody production reached 1.3 ± 0.09 g L⁻¹, almost doubling the yield obtained in the stirred-tank reactor. The improved outcomes observed in the innovative bioreactor could be associated with the distinct operating and hydrodynamic conditions established by the system configuration. Nevertheless, further studies are needed to better define the operational limits of the system and optimize process conditions. Future investigations should also assess critical quality attributes, including glycosylation patterns, aggregation, fragmentation, and host cell protein contamination, as well as evaluate the performance of the bioreactor at larger scales.

**Graphical abstract:**

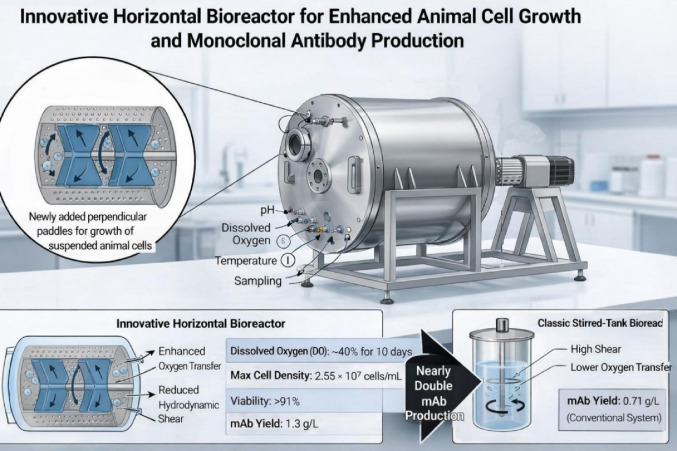

**Supplementary Information:**

The online version contains supplementary material available at 10.1007/s10616-026-01036-1.

## Introduction

Nowadays, medicine is increasingly moving toward personalized approaches tailored to specific disease characteristics (Malik and Ghatol 2021). Monoclonal antibodies (mAbs) represent the largest class of biopharmaceutical proteins. Owing to their structural and functional versatility, mAbs are employed in a broad range of therapeutic applications, ranging from the treatment of cancer (Zahavi and Weiner 2020) and autoimmune diseases (Elliott et al. 2008) to the management of viral infections (Chahine and Durham 2021) and the prevention of tissue rejection (Eskandary et al. 2019). Indeed, mAbs have significantly improved treatment strategies for autoimmune diseases, and several mAbs have been developed for the treatment of various neoplastic diseases (Castelli et al. 2019).

This broad range of applications, combined with their enhanced specificity and selectivity compared with conventional therapies, has driven significant interest in the production of recombinant biotherapeutic proteins (Jain and Kumar 2008). In 2024, the U.S. Food and Drug Administration (FDA) approved 16 biologic drugs, representing approximately 32% of all approved biologics, 13 of which were mAbs (Martins et al. 2025).

These recombinant proteins are produced in heterogeneous host cells using transgene technology (Li et al. 2018). Chinese Hamster Ovary (CHO) cells among the most widely used mammalian cell lines in the biopharmaceutical industry for the production of therapeutic proteins, particularly monoclonal antibodies (mAbs), recombinant enzymes, and cytokines. Since their introduction in the 1980s, CHO cells have become the gold standard for biologic manufacturing due to their ability to perform complex post-translational modifications, such as glycosylation, which are essential for the efficacy, stability, and safety of many protein-based drugs. Indeed, among the best-selling biopharmaceuticals worldwide in 2019, seven out of eight were monoclonal antibodies produced in CHO cells, mainly due to the ability of these cells to achieve accurate glycosylation and protein folding processes comparable to those of human cells (Yang et al. 2022).

CHO cells are favoured for several reasons: they grow efficiently in suspension cultures, adapt readily to serum-free and chemically defined media, and exhibit relatively high tolerance to genetic manipulation and scale-up conditions. These characteristics make them highly suitable for industrial-scale production under Good Manufacturing Practice (GMP) conditions (Du and Webb 2011; Ritacco et al. 2018).

Processes enabling this type of cell growth are still under continuous improvement, and the development of novel bioreactors and production methods remains a major challenge. Current systems are unable to fully meet the increasing demand, and production costs remain high, making mAbs one of the most expensive classes of therapeutics currently in use (Kelley 2009).

Several types of bioreactors, including bubble column bioreactors, fluidized bed bioreactors, and packed bed bioreactors, have been developed; however, stirred-tank bioreactors are the most widely used systems today. Traditionally, these bioreactors consist of cylindrical tanks with an H/D ratio typically ranging from 3 to 5 and are equipped with an impeller for homogenizing the culture medium and a sparger for supplying oxygen to the cells (Najafpour 2007).

These bioreactor types offer several advantages, making them ideal for large-scale production, where consistency and efficiency are key factors. High-performance bioreactors incorporating perfusion systems, in which continuous medium exchange occurs to provide long-term culture stability and high cell density, have also been developed (Jyothilekshmi and Jayaprakash 2021).

However, the main bottleneck limiting the maximal efficiency of these traditional bioreactors is the availability of dissolved oxygen (DO) throughout the entire fermentation cycle, due to its low solubility in water. As culture density increases, oxygen demand rises, often making oxygen transfer a rate-limiting factor in bioreactor systems. Inadequate oxygen supply can lead to hypoxic stress, resulting in reduced cell growth, decreased protein expression, and shifts toward undesirable metabolic pathways (e.g., increased lactate production) (Wenger et al. 2015; Gomes et al. 2016).

To address this limitation, oxygen is typically supplied through increased agitation or gas sparging; however, these approaches involve trade-offs, including increased hydrodynamic stress (shear stress), which can be detrimental to CHO cells (Garcia-Ochoa et al. 2010). Unlike microbial systems, mammalian cells lack rigid cell walls and are sensitive to mechanical damage; therefore, excessive shear stress can disrupt cell membranes, induce apoptosis, and reduce protein production (Hu et al. 2011).

To mitigate these effects, an innovative bioreactor was developed by Eco-Sistemi srl in the present study. The prototype bioreactor was designed to ensure adequate oxygen supply to the cultured cells by increasing the liquid surface area exposed to the headspace, thereby enabling more efficient gas exchange even at low rotational speeds.

To evaluate the performance of the system, a stably transfected CHO cell line capable of producing a mAb was cultured in the innovative bioreactor. For this purpose, cell growth, viability, and recombinant protein yield were monitored throughout the process and compared with those obtained using a traditional bioreactor.

## Materials and methods

### Description of the bioreactor prototype and its innovative principle

**Fig. 1:**
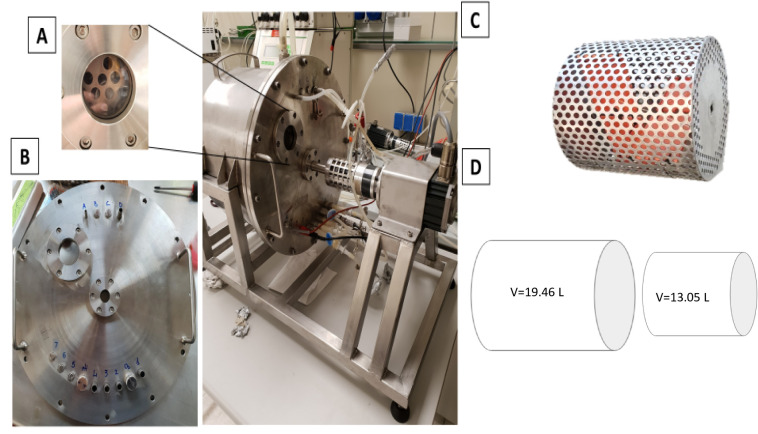
Pictures of the prototype: **A**) shows the prototype in its working position, with a zoomed view of the small window that allows visual inspection inside the vessel; **B**) shows the ports on the flange used for physico-chemical parameters monitoring/control and sampling; **C**) shows the perforated basket equipped with two perpendicular paddles for suspension cell growth; **D**) shows the cylindrical volumes of the vessel (left) and the perforated basket (right)

The bioreactor prototype (Eco-Sistemi srl, Italy), shown in Fig. 1, was originally designed for bacterial growth for applications in wastewater treatment. The prototype consists of a horizontal chamber (vessel) with a length of 24 cm and a diameter of 32 cm, coupled to a slow-rotating perforated basket (length and diameter: 22.3 cm and 27.3 cm, respectively). The basket contains two perpendicular paddles, which ensure efficient homogenization of the liquid culture. The vessel is equipped with several inlet and outlet ports and probe slots for monitoring temperature, pH, foam formation, and pO₂. Once introduced into the vessel, the culture medium is maintained at the desired temperature through the circulation of heating/cooling water within a jacket surrounding the vessel.

The main innovative principle underlying this prototype involves increasing the liquid surface area exposed to the headspace, thereby promoting gas exchange at low rotational speeds. The internal volume of the vessel is 19.46 L, with maximum and minimum working volumes of 6 and 0.75 L, respectively.

### Measurement of the volumetric mass transfer coefficient by the static gassing-out method

The volumetric mass transfer coefficient (k_L_a) is a parameter that describes the rate at which O₂ is transferred between the gas and liquid phases. The k_L_a was determined using an experimental method in which the medium inside the bioreactor is initially deoxygenated. Air is then introduced at a constant flow rate, and the dissolved oxygen concentration is monitored over time. This method, known as the static gassing-out method, is one of the most widely used approaches for k_L_a evaluation (Vanags and Suleiko 2020) The method can be described by the following Eq. 1, proposed by Wise (Wise 1951).

1 $$\:OTR\:=\frac{dC}{dt}={k}_{L}a\:\left({C}^{*}-{C}_{L}\right)$$

where,

OTR is oxygen transfer rate.

C^*^ is the saturated dissolved oxygen concentration.

C_L_ is the current concentration of dissolved oxygen in the medium.

By integration of the Eq. 1 with C_L_ (t_0_)=C_0_ the Eq. 2 is obtained:

2 $$\:\mathrm{ln}\frac{{(C}^{*}-{C}_{t})}{{(C}^{*}-{C}_{0})}={-k}_{L}a*t$$

**Fig. 2:**
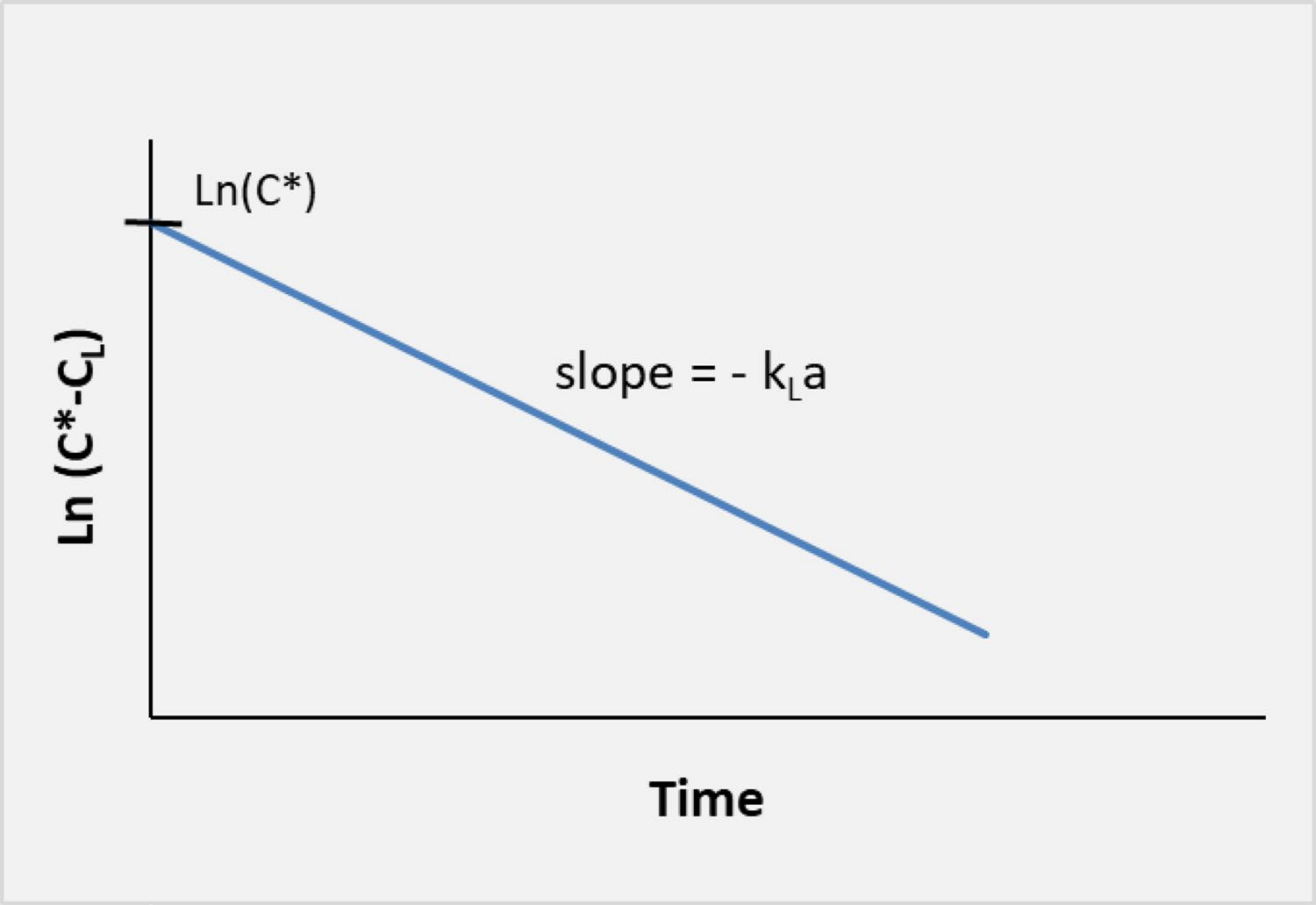
Graphic of Ln(C*-C_L_) plot on time to determine k_L_a value by the gas-out static method

Plotting ln(C*-*C*_*L*_) against time we have a straight line where the slope is equal to -k_L_a, as shown in Fig. 2.

To evaluate the k_L_a, two different experiments were performed using both traditional and innovative bioreactors with initial working volumes of culture medium of 0.8 L and 2.6 L, respectively. The traditional bioreactor used for comparison consisted of a stirred vessel with a diameter of 13 cm, equipped with two standard six-blade Rushton impellers (diameter: 0.04 m).

Phosphate-Buffered Saline (0.5 M, pH 7.2) was used as the medium at a temperature of 37 °C. In both systems, nitrogen was sparged into the medium until the dissolved oxygen concentration reached zero (C₀). The nitrogen flow was then stopped, and the nitrogen in the headspace was replaced by continuous air flow. For both bioreactors, the volume of air required was approximately twice the volume of the respective headspaces.

At the beginning of the experiment, a ring sparger located at the bottom of the traditional bioreactor vessel sparged air into the medium at a constant flow rate of 0.1 slpm, while the agitation speed was maintained at 180 rpm. For the innovative bioreactor, air was introduced into the headspace at a constant flow rate of 0.1 slpm, and the drum rotation speed was maintained at 7 rpm (the minimum speed of the overhead stirrer). For both experiments, the dissolved oxygen (DO) percentage was measured using an O₂ probe (InPro 6100 Series, Mettler Toledo, Switzerland) connected to the Biostat^®^ B unit, and DO values were recorded using MFCS/WIN software (version 2.1).

## Hydrodynamic characterization of the two bioreactor systems

The hydrodynamic parameters of the two bioreactor configurations were quantified using standard engineering correlations with water as the medium at 37 °C and 1 atm. The Reynolds number (Re) and the impeller tip speed (ν_tip_) were calculated using the Eqs. 3 and 4, respectively

3 $$\:Re=\frac{\rho\:N{D}^{2}}{\mu\:}$$

4 $$\:{v}_{\mathrm{tip}}=\pi\:DN$$

where,

ρ is the liquid density (993 kg·m⁻³), N the rotational speed (s⁻¹; rpm/60), D the characteristic diameter (impeller diameter for the traditional bioreactor and drum diameter for the innovative bioreactor), and µ the dynamic viscosity (0.000694 Pa·s).

For stirred-tank reactors, the specific power input was estimated using the classical Rushton turbine correlation (Kaiser et al. 2017), according to Eq. 5:

5 $$\:{\frac{P}{V}}_{\mathrm{tra}}=\frac{{N}_{P}\rho\:{N}^{3}{D}^{5}}{V}$$

where.

N_P_ is the impeller power number, which for a 6-blade Rushton turbine in standard turbulent conditions is equal to 5, V the working volume, and the remaining symbols are as defined above.

For the innovative bioreactor with a rotating drum, the specific power input was estimated from the experimentally determined oxygen transfer coefficient using a Van’t Riet–type gas–liquid mass transfer correlation (van’t Riet 1979).

Although this correlation was originally developed for conventional stirred systems, a first-order comparison of the hydrodynamic conditions between the two bioreactor configurations could be established by assuming that mixing in both systems is predominantly driven by rotating elements.

This assumption is justified by the low aeration rates typically used in mammalian cell cultures, under which the contribution of gas flow to the power number is expected to be limited (Gabelle et al. 2011). The Van’t Riet correlation could be reduced to a power-law dependence of k_L_a on volumetric power input, with the gas velocity term incorporated into the empirical constant (Eq. 6).

6 $$\:{k}_{L}a=K{\left(\frac{P}{V}\right)}^{\alpha\:}$$

Therefore, an empirical relationship (Eq. 7) between the volumetric power input (*\:P/V*) and the volumetric mass transfer coefficient ($\:{k}_{L}a$) was used to estimate the specific power input (P/V_inno_) for innovative bioreactor

7 $$\:\frac{{P/V}_{inno}}{{P/V}_{tra}}\:={\left(\frac{{k}_{L}{a}^{\mathrm{inno}}}{{k}_{L}{a}^{\mathrm{tra}}}\right)}^{1/\alpha\:}$$

with $\:\alpha\:=0.5$, a typical exponent for coalescent media in turbulent mixing.

The hydrodynamic parameters were computed using the measured operational conditions (7 and 180 rpm for the innovative and traditional bioreactor, respectively) and the experimentally determined k_L_a values.

Hydrodynamic shear conditions were estimated from the local energy dissipation rate under turbulent flow conditions at 37 °C. The specific power input (P/V) for each bioreactor was first determined as described above. The average energy dissipation rate was then calculated according to Eq. 8:

8 $$\:\epsilon\:=\frac{P}{\rho\:\:V}$$

Assuming isotropic turbulence, the characteristic shear rate was estimated by Eq. 9:

9 $$\:\dot{\gamma\:}={\left(\frac{\epsilon\:}{\nu\:}\right)}^{1/2}e$$

where.

ν is the kinematic viscosity of water (0.70 × 10⁻⁶ m²·s⁻¹). The corresponding shear stress was calculated by Eq. 10:

10 $$\:\tau\:=\mu\:\dot{\gamma\:}$$

where $\:\mu\:\:$is the dynamic viscosity.

These shear estimates were used to compare the hydrodynamic environment of the rotating drum with that of a traditional bioreactor.

### Cho cells and expansion

A glutamine synthetase Chinese hamster ovary (GS-CHO) cell line producing a recombinant monoclonal antibody (mAb) was used in this study.

Prior to bioreactor inoculation, a vial of GS-CHO cells was retrived from the cell bank, thawed, and aseptically cultured in a 250 mL flask containing 80 mL of CD FortiCHO™ medium (Life Technologies Europe BV, The Netherlands) in a shaking CO_2_ incubator (Thermo Scientific, model 8000DH) at 37 °C with a shaking frequency of 110 rpm, a relative humidity of 70%, and 5% (v/v) CO_2_ (Warner et al. 2015).

The culture was subcultured every 3–4 days until bioreactor inoculation. The contents of the Erlenmeyer flasks were centrifuged (700 rpm, 8 min) to remove the spent medium, resuspended in fresh medium, and reseeded into Erlenmeyer flasks at a high seeding density. The cultures were then incubated in the shaker incubator (under the same conditions described above) for 24 h. Afterwards, the cells were inoculated into the bioreactor prototype and a 2 L Sartorius bioreactor.

### Culture conditions in bioreactors

In this study, three runs were performed using the new-concept bioreactor prototype described above and a 2 L stirred bioreactor equipped with a Rushton impeller (B. Braun Biotech International, Germany).

Both systems were controlled by a Biostat^®^ B benchtop unit (B. Braun Biotech International) equipped with a gas mixing module and an overhead stirrer (Heidolph RZR 2102 Control, Germany) for mixing. During the culture, pH was automatically controlled at 7.15 by the addition of either 1.5 M NaHCO_3_ or 1 M HCl, while the temperature was set at 37 °C. For both bioreactors, 7% CO_2_ was supplied to the headspace at a flow rate of 0.1 slpm.

The dissolved oxygen (DO) concentration was maintained at 40% of air saturation by increasing the stirrer speed and pulsing pure O_2_. The agitation speed was increased stepwise in the range from 180 to 250 rpm in the traditional bioreactor and from 7 to 15 rpm in the innovative bioreactor. Oxygen was supplied by a gas mixer into the liquid phase of the traditional bioreactor through a ring sparger located at the bottom of the vessel, whereas in the innovative bioreactor oxygen was introduced into the headspace instead of air.

CD FortiCHO™ medium supplemented with 1% penicillin-streptomycin (GE Healthcare Bio-Sciences) was introduced aseptically by filtration, with an initial working volume of 2.6 and 0.8 L in the new prototype and the Sartorius bioreactor, respectively. Both bioreactors were inoculated at a cell density of 0.5 × 10⁶ cells mL⁻¹, and cells were cultured in fed-batch mode for 10 days.

CHO cells were sampled from the bioreactors every 24 h, and cell density and viability were measured using a trypan blue exclusion assay with a Bürker chamber and a Nikon Eclipse E400 microscope. Glucose and lactate concentrations were determined using HPLC. Based on daily glucose consumption, CD EfficientFeed™ C AGT™ Nutrient Supplement (Gibco™, Thermo Fisher Scientific, USA) and glucose were added to maintain a concentration of 4 g/L during the culture process. Based on the total glucose supplied during the entire culture and the final lactate concentration, the apparent conversion of glucose to lactate was estimated for both bioreactors.

### Determination of monoclonal antibody (mAb) Titer and Glucose and Lactate Concentrations

Samples were filtered through a 0.22 μm Millipore filter into 2 mL vials and analyzed using an Agilent 1200 Series HPLC system (Agilent Technologies) equipped with a degasser module (G1379B), a binary pump (G1312B), an autosampler (G1367B), a column compartment (G1316A), a UV-Vis detector (G1314B) and a refractive index detector (RID, G7162A).

To determine the concentration of the aspecific monoclonal antibody present in the culture medium of the transfected cell line, chromatographic separation was performed using a pre-packed affinity column functionalized with Protein A (POROS™ A 20 μm Column, 4.6 × 100 mm, 1.7 mL). The injection volume was 20 µL, sodium phosphate buffer (0.15 M, pH 7.0) was used as the mobile phase with a flow rate of 0.7 mL min^− 1^ under isocratic conditions. The column was maintained at room temperature, and the run time was 20 min. Detection was performed using a UV–Vis detector at a wavelength of 210 nm. A six-point calibration curve was generated using human serum IgG (Sigma-Aldrich, I4506) as the standard.

To determine glucose and lactate concentrations in the culture medium, chromatographic separation was performed using an Agilent Hi-Plex H analytical column (7.7 × 300 mm, 8 μm) maintained at 60 °C. The run time was 15 min, and the injection volume was 20 µL. The mobile phase consisted of 0.005 M sulfuric acid delivered at a flow rate of 0.7 mL min⁻¹ under isocratic conditions. Detection was performed using a refractive index detector (RID). Calibration curves consisting of at least five concentration points (0.05–10 g/L) were generated for both glucose and lactate standards, showing excellent linearity (R² ≥ 0.9999).

All data were collected and analyzed using the software OpenLAB CDS Chemstation Edition Rev. C.01.10(201).

### Data analysis

Each experimental condition was evaluated in triplicate. Each sample was analyzed three times, and the mean values of CHO cell density and mAb titer were calculated. All data are expressed as mean ± standard deviation (SD). The effects of bioreactor type, culture time, and their interaction on mAb production were assessed using a two-way repeated-measures ANOVA.

Pairwise comparisons at each time point were further evaluated using independent t-tests with Bonferroni correction to account for multiple testing. In addition, cell growth in the different bioreactors was modeled using a nonlinear Gompertz function to estimate key growth kinetic parameters. All analyses were performed using R software (version 4.5.1).

## Results

### Volumetric mass transfer coefficient

**Fig. 3:**
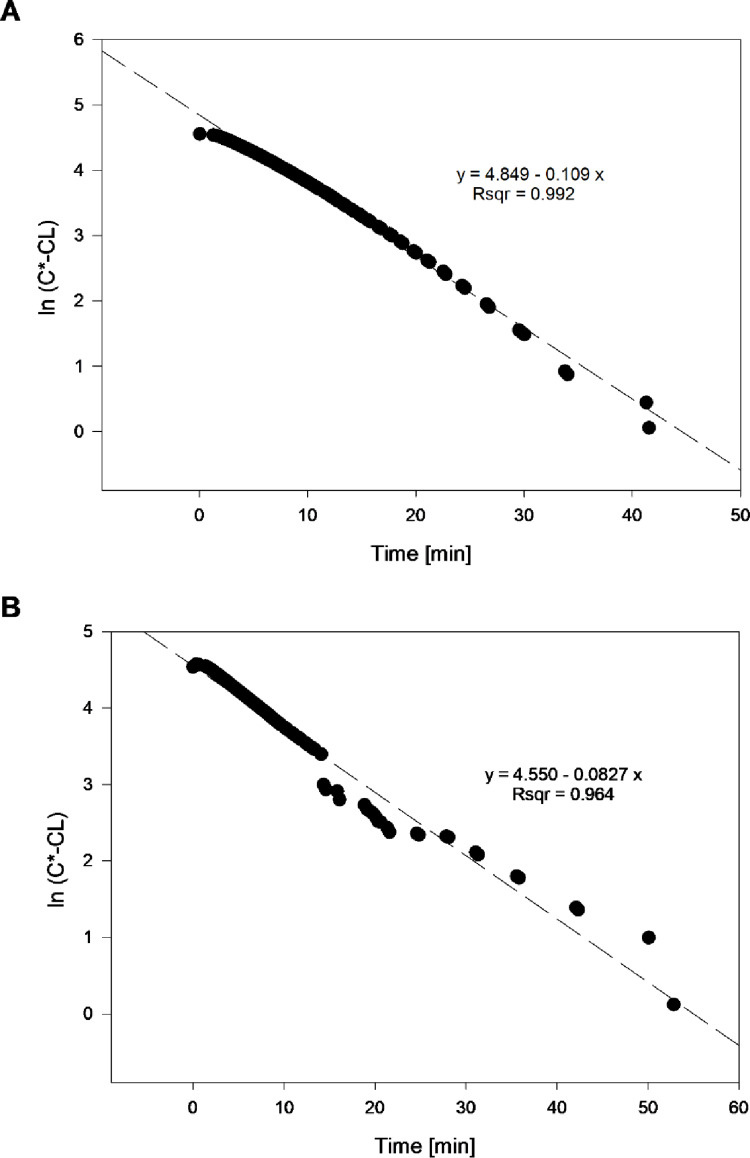
Plot of ln (C*-C_L_) versus time (min) recorded for the innovative bioreactor (**A**) with a rotation speed of 7 rpm and the traditional bioreactor (**B**) at 180 rpm with air flow of 0.1 slpm for both

Experimental analysis to determine the oxygen transfer rate (OTR) in both bioreactor systems under constant conditions was performed using the static gassing-out method. Figure 3 shows plots of ln(C* − C_*L*_*)* versus time (min) for the innovative and traditional bioreactors, respectively. As shown, the negative slope of the straight line, which corresponds to the volumetric mass transfer coefficient (− k_L_a), was higher in the innovative bioreactor (0.1087 min⁻¹) than in the traditional bioreactor (0.0827 min⁻¹).

### Hydrodynamic characterization of the two bioreactor systems

To enable a consistent comparison between the two cultivation systems, an engineering characterization of both the innovative rotating‑drum bioreactor and the conventional stirred‑tank bioreactor was performed.

During operation, the drum rotates at 7–15 rpm, corresponding to tip speeds of 0.10–0.21 m·s⁻¹ and Reynolds numbers in the range of 1.14 × 10^4^ to 2.45 × 10^4^. These values remain substantially lower than those of conventional Rushton-stirred tanks operating between 180 and 250 rpm, which exhibit higher tip speeds (0.38–0.52 m·s⁻¹) and Reynolds numbers of 6.54 × 10^3^ to 8.96 × 10^3^. Despite both systems operating in the turbulent regime, the rotating drum achieves turbulence at markedly lower rotational speed due to its larger characteristic diameter.

Under the operating conditions used for experimental k_L_a determination, the conventional bioreactor exhibited a specific power input of 34.31 W·m⁻³ at 180 rpm, as calculated using the Rushton turbine correlation. For the innovative bioreactor, the specific power input (P/V), estimated from k_L_a correlation using a Van’t Riet–type equation at 7 rpm, was approximately 59.29 W·m⁻³.

Hydrodynamic shear conditions at these resulting specific power input (P/V), estimated from the bulk energy dissipation rate under turbulent flow, corresponded, for the traditional bioreactor, to an average shear stress of 0.16 Pa, consistent with conventional laboratory-scale STRs (Rivas et al. 2014; Kwon et al. 2024; Lemire et al. 2026). In contrast, for the innovative bioreactor at 7 rpm the average shear stress was 0.21 Pa.

### Comparison of viable cell density (VCD), viability, and dissolved oxygen

Production fed-batch cultures were performed in bioreactors at a seeding density of 0.5 × 10⁶ cells mL⁻¹ and a duration of 10 days. The final culture volume, including the amounts of feed, base, and antifoam added, corresponded to 96.15% and 87.5% of the initial volume in the innovative and traditional bioreactors, respectively.

**Fig. 4:**
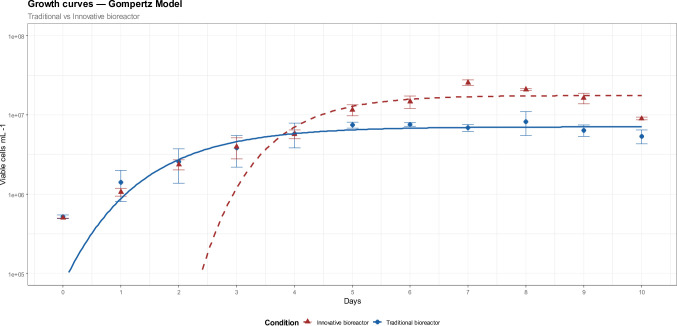
Viable cell density (VCD) profiles of CHO cell cultures grown in an innovative prototype and a traditional bioreactor. Data represent the mean ± SD of three independent replicates. Symbols indicate experimental measurements, while solid and dashed lines represent Gompertz model fits for the traditional and innovative systems, respectively

As shown in Fig. 4, both processes exhibited a progressive increase in viable cell density (VCD) over time, with comparable behaviour during the initial phase (days 0–4), indicating similar adaptation kinetics. From day 4 onward, the innovative bioreactor showed a markedly enhanced proliferation rate, reaching a peak VCD of 2.55 × 10⁷ cells mL⁻¹ on day 7, more than threefold higher than that observed in the traditional system. These results indicate a substantial difference in cell growth performance between the two bioreactor configurations under the tested operating conditions.

A decline in VCD was observed after the peak in the innovative bioreactor based on conventional cell counting. However, this decrease did not reflect an actual loss of viable cells. From day 8 onward, the presence of numerous cell aggregates (clumps) was detected, leading to a substantial underestimation of cell density, as standard counting methods are unable to accurately quantify aggregated cells.

To overcome this limitation, samples were subjected to enzymatic dissociation prior to counting using 0.5% trypsin-EDTA at 37 °C for 8 min. As shown in Figure S1, this treatment revealed significantly higher VCD values at later time points, reaching approximately 4.1 × 10⁷ cells mL⁻¹ on day 10, compared with markedly lower values obtained from untreated samples. These results confirm that the apparent decline in VCD is primarily attributable to cell aggregation rather than to a decrease in cell viability.

This aggregation effect also influenced the quality of the growth model fitting. The lower coefficient of determination observed for the innovative bioreactor (R² 0.7455 vs. 0.8943 for the traditional system) can be attributed to the inability of the Gompertz model to account for measurement artifacts associated with clump formation during the late phase of culture. When considering only the 0–7 day interval, prior to significant aggregation, the model fit is expected to improve substantially. A detailed summary of the fitted parameters is provided in Supplementary Data (Table S1).

**Fig. 5:**
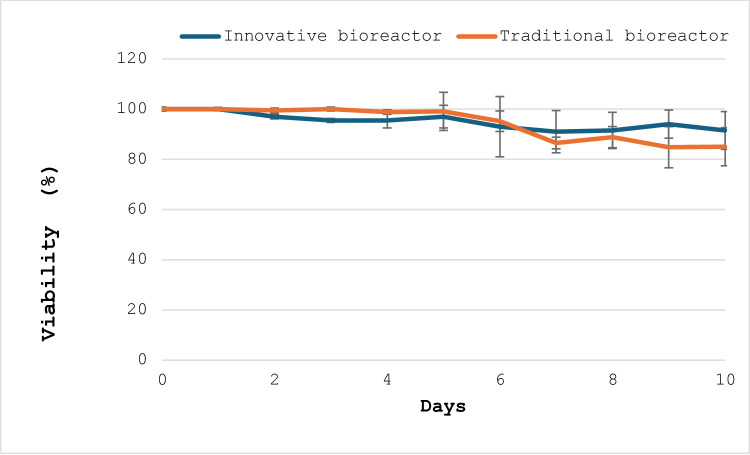
Viability percentage recorded during CHO cell culture performed using an innovative prototype and a traditional bioreactor. The data represent average values of three replicate

**Fig. 6:**
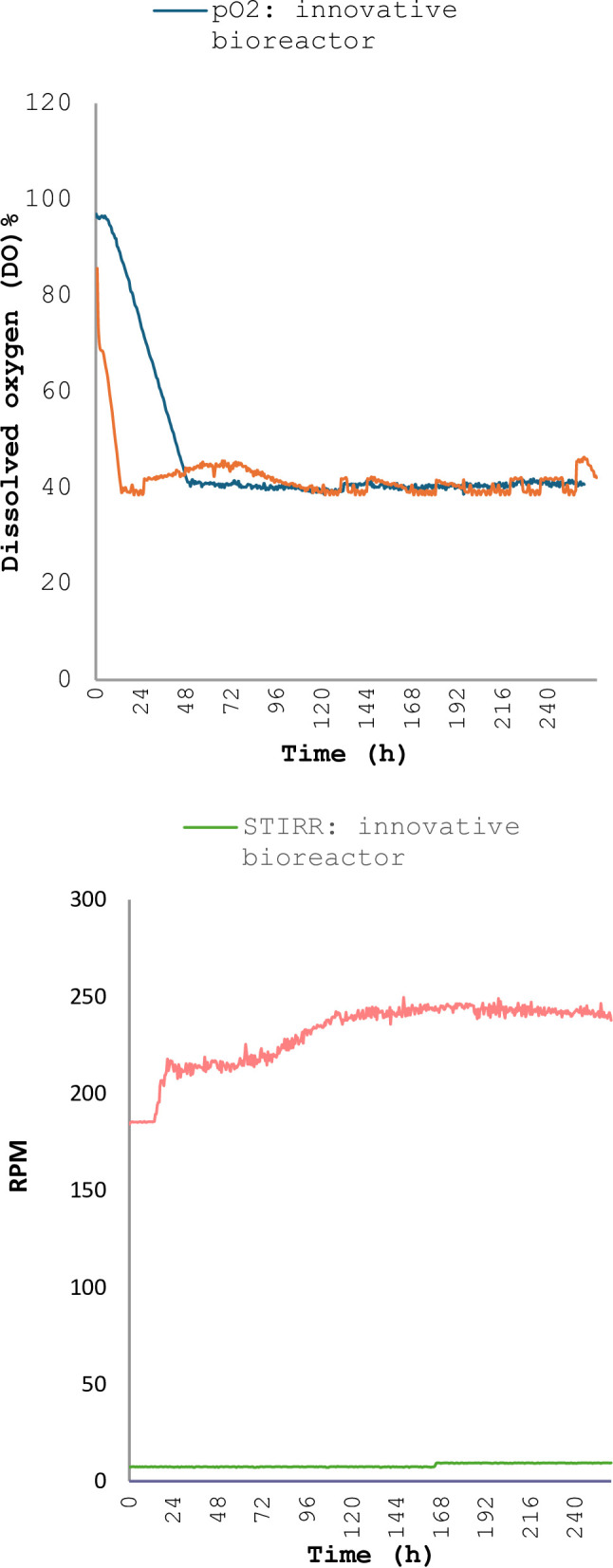
Graphs showing the monitoring of dissolved oxygen levels (a) and stirrer speed (b) recorded in the innovative prototype and traditional bioreactor during cell culture

Furthermore, significant differences were observed between the two processes in terms of viability and dissolved oxygen, as shown in Figs. 5 and 6a. The high cell viability during the culture cycle in the innovative bioreactor is noteworthy, reaching 91.7% at day 10, compared with 85.0% in the traditional bioreactor. The dissolved oxygen concentration in culture medium reached approximately 40% after about 12 h and 48 h in the traditional and innovative bioreactors, respectively. As shown in Fig. 6b, in the traditional bioreactor the stirrer speed had already started increasing after 12 h.

### mAb productivity in two different bioreactors

**Fig. 7:**
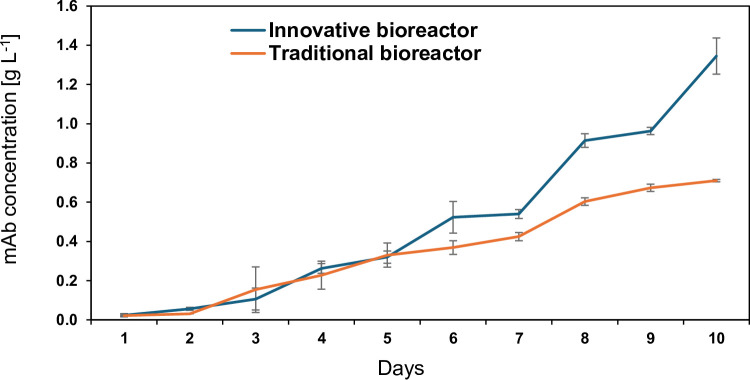
Time course of mAb titer recorded during CHO cell culture performed using an innovative prototype (blue line) and a traditional bioreactor (orange line). Data represent mean values of three replicate runs and error bars indicate the standard deviation between replicates

The mAb production, shown in Fig. 7, reveals a continuous increase over time and, despite the use of the same cell line, clear differences in yields were observed between the two bioreactors. In particular, using the innovative bioreactor, a titer of 1.3 ± 0.09 g L⁻¹ was achieved at day 10. In contrast, a titer of 0.71 ± 0.006 g L⁻¹ was obtained when the cell culture was carried out using the traditional bioreactor at the same time. Moreover, from the graph reported in Fig. 7, it is evident that mAb production did not reach a plateau at the final day, regardless of the bioreactor considered. In fact, as reported above, cell viability at the final day was still high, especially in the innovative bioreactor (91.7%).

A two-way repeated measures ANOVA revealed a significant effect of both time and bioreactor type, as well as a significant time × bioreactor interaction, indicating that the production dynamics differ between the two systems. Post-hoc analyses (see Supplementary Data, Table S2 and S2.1) using independent t-tests with Bonferroni correction showed that differences between the bioreactors became statistically significant only at later stages of the culture (days 8–10), whereas no significant differences were observed during the early and intermediate phases.

#### Glucose consumption and lactate level

**Fig. 8:**
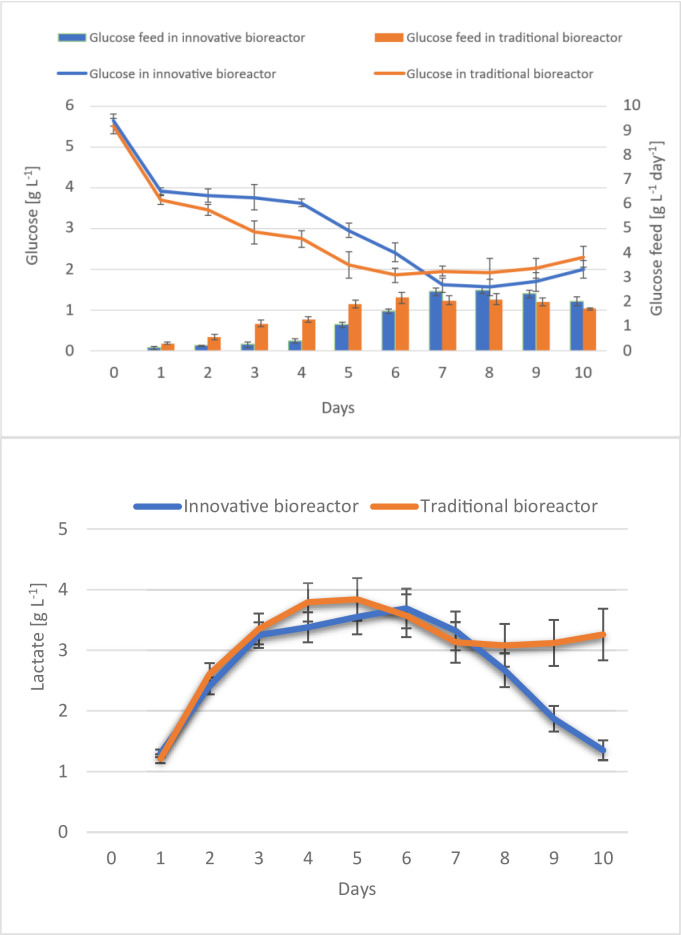
Glucose and lactate concentration in CHO cell cultivations performed using an innovative prototype bioreactor and a traditional bioreactor. Data represent mean values of three replicate runs, and error bars indicate the standard deviation between replicates

As shown in Fig. 8, glucose concentration decreased progressively in both systems, with the innovative bioreactor maintaining slightly higher residual glucose levels throughout the culture. Glucose feed was gradually increased from day 0 to day 5 and then stabilized, reaching comparable cumulative amounts in both bioreactors by day 10. While, lactate levels in the culture broth increased during the first 3 days, reaching up to 3–4 g L⁻¹ depending on the fermentation run. Levels then reached a plateau and decreased during the final days. This indicates that, after the initial culture phase, cells begin to utilize lactate as an energy source. A significant difference in lactate concentration was observed between the two bioreactors; in particular, a lower lactate level of 1.35 g L⁻¹ was achieved with the innovative bioreactor at day 10, while the traditional bioreactor reached a value of 3.26 g L⁻¹.

As shown in Table 1, despite a similar total glucose supply in the innovative and traditional bioreactors, lactate accumulation was lower in the innovative bioreactor (1.35 g·L⁻¹ vs. 3.26 g·L⁻¹). This resulted in a reduced apparent glucose-to-lactate yield compared with the traditional system.

**Table 1:** Total glucose consumption and lactate production in the two bioreactor systems

Parameter	Innovative	Traditional
Total glucose supplied (g·L⁻¹)	12.73 ± 3.53	15.07 ± 2.91
Final lactate concentration (g·L⁻¹)	1.35 ± 0.16	3.26 ± 0.42
Apparent yield (w/w)	0.106	0.216

Data reported represent mean values ± standard deviation

## Discussion

In this study, a novel bioreactor configuration was evaluated and compared with a traditional stirred-tank system. Particular emphasis was placed on assessing k_L_a, alongside cell growth and productivity, while the hydrodynamic characteristics of the two bioreactors were also evaluated. Improvement of volumetric gas-liquid mass transfer coefficient can be realized by increasing the gas–liquid mass transfer coefficient term ‘k_L_’ and increasing the gas–liquid interfacial area term ‘a’ (Warner et al. 2015; He et al. 2022). The design of the bioreactor in combination with physico-chemical and rheological characteristics of the culture medium is directly connected with volumetric gas-liquid mass transfer coefficient (K_L_a) (Vanags and Suleiko 2020).

The k_L_ values are known to be affected by a multitude of factors such as gas diffusivity, density and viscosity of the liquid medium, and gas–liquid affinity, which make it difficult to increase. Instead, the gas–liquid interfacial area term ‘a’ is rather relatively easy to be increased and in the innovative bioreactor it was achieved by slow rotation of perforated basket that allows the formation of a flowing film of liquid.

In fact, as visible in Fig. 6a, the dissolved oxygen concentration in culture medium considerably decreased in the traditional bioreactor respect to innovative bioreactor even though the cell density was comparable among the two systems. Furthermore, the innovative system maintained the DO level at 40% during all the 10 days of cell culture cycle, with just automatic addition of 0.1 L min^− 1^ of O_2_ in the headspace and slowly rotation speed of 7–15 rpm.

This is consistent with improved mass transfer via a rotating basket that generates a thin liquid film and increases the gas-liquid interfacial area, even at low rpm. The oxygen transfer efficiency, observed through the static gassing out analysis, was higher in the innovative system at minimal rate stirring (7 rpm), with a k_L_a of 0.1087 min⁻¹ compared to 0.0827 min⁻¹ in the traditional bioreactor at 180 rpm.

The value of 0.1087 min⁻¹ (6.52 h⁻¹) represents a moderate oxygen transfer rate, which is in the range of 1 and 10 h^− 1^, typically achievable under low gas flow rates and gentle agitation, which are ideal conditions for shear-sensitive animal cells (Kaiser et al. 2011).

Maintaining adequate gas mass transfer and supplying O_2_ are essential for supporting both cell metabolism and protein production, especially as biomass increases (Xu et al. 2017). Although, in mammalian cell cultures, dissolved CO₂ plays a critical role in determining culture conditions and performance, particularly through its interaction with the bicarbonate buffering system and its direct effect on pH (Klinger et al. 2020).

It should be noted that no direct measurements of a CO₂ specific mass transfer coefficient (k_L_a,_CO₂_) were performed in this study. However, it is well established that conventional scale-up and characterization strategies based on oxygen k_L_a do not necessarily account for CO₂ accumulation or removal, which can significantly affect pH and process performance. This interpretation is further supported by the general understanding that CO₂ transfer and removal are governed by distinct mechanisms compared to oxygen, including higher solubility and bubble saturation effects (Nickel et al. 2024).

In our study, without CO₂ supplementation in the headspace (5–7%), a relatively rapid increase in medium pH was observed only in the innovative bioreactor. This behavior is compatible with a possible removal of CO₂ from the carbonate buffering system and reflects the known dependence of pH on CO₂ partial pressure in mammalian cell culture media (Zhao et al. 2023). Although this difference does not constitute direct evidence of enhanced CO₂ removal and should therefore be interpreted with caution.

Essentially, oxygen transfer, carbon dioxide stripping and mixing parameters are all intimately connected and need to be considered in an integrated way to bioreactor design with aim improving mass transfer without excessive damage to the cells caused by shear stress (Nienow 2015; Strobl et al. 2021).

In this context, an empirical evaluation of the hydrodynamic conditions in the two bioreactors was performed to provide an estimation of the shear stress. It should be noted that commonly adopted methodologies for hydrodynamic and shear characterization could be not fully applicable to the innovative bioreactor configuration due to its distinct geometry and mixing mechanism.

Nevertheless, an attempt to estimate and compare hydrodynamic conditions was carried out using well-established correlations, under the assumption that agitation in both systems is ultimately driven by rotating elements. From these characterizations, it clearly appears both systems operating in the fully turbulent regime. However, the rotating drum could achieve turbulence at markedly lower tip speeds (0.10 m s^− 1^) compared to traditional bioreactor with the Rushton turbine (0.47 m s^− 1^). This phenomenon is driven by the larger characteristic diameter of the drum. As a result, the rotating drum maintains efficient gas–liquid mass transfer even at low rotational speeds (Duan et al. 2021).

The specific power input per unit volume (P/V) was comparable between the two systems, for innovative at 7 rpm and traditional at 180 rpm were 59.29 and 34.31 W m^− 3^, respectively.

The corresponding average shear stresses derived from the bulk energy dissipation rate (ε_avg_) were 0.21 and 0.16 Pa for the innovative and traditional bioreactor, respectively. However, average shear stress does not capture the heterogeneous nature of hydrodynamic stress in stirred systems, where energy dissipation is strongly localized near impeller blades (Jain et al. 2025). Using geometric correlations for local energy dissipation (ε_local_ = ε_avg_ × D_reactor_/D_impeller_) (Hortsch and Weuster-Botz 2010), ε_local_ values of 0.25 and 0.52 Pa were obtained for the innovative and traditional bioreactor, respectively.

However, the potential biological implications of local stress distributions could not be directly assessed in the present study and therefore require further investigation. To properly interpret the biological impact of these physical stresses on suspended CHO cells, these values must be benchmarked against the mechanical tolerance thresholds reported in the literature. Hydrodynamic studies indicate that the critical threshold triggering acute cell lysis and a drastic drop in viability for suspended CHO cells is approximately 32.4 ± 4.4 Pa (Neunstoecklin et al. 2015). Therefore, both examined bioreactors operate well below the limit of direct mechanical lethality. Although the local shear stress of 0.52 Pa, estimated for the traditional bioreactor, is not a mechanically dangerous shear level, it could be high enough to activate cellular stress responses, including early apoptotic signaling. This interpretation is consistent with the hydrodynamic stress framework described by Kenmoku et al. 2025. The authors demonstrate that shear stress is a primary driver of performance loss in CHO cultures, with clear reductions in cell growth and product titer occurring well below the levels traditionally considered damaging. The study further highlights that mammalian cells respond to hydrodynamic stress in a dependent manner and that even relatively low shear values can trigger biologically significant stress responses.

Onset of sublethal responses, metabolic alterations (such as increased glucose consumption and lactate production), or a partial inhibition of recombinant protein synthesis has been documented under persistent hydrodynamic stresses (Tanzeglock et al. 2009).

A substantial decrease in recombinant protein productivity due to shear stress was presented for protein free media supplemented with Pluronic, where specific productivity decreased rapidly from 100.4% at 0.005 Pa to 65.8% at 0.10 Pa. The decrease continued, although at a slower rate, from 0.10 to 0.80 Pa (Keane et al. 2003).

In addition to this complex landscape of purely fluid dynamic stresses, a further critical mechanical risk factor exclusive to traditional systems must be considered: the phenomenon of bursting bubbles at the free liquid surface (Walls et al. 2017). In stirred tank bioreactors, maintaining adequate DO levels requires continuous sparging. When the system operates at higher volumetric gas flow rates, the intensive generation of bubbles drastically amplifies the risk of cellular damage (Chaudhary et al. 2020). In the literature, it has long recognized that the most damaging kinematic event for suspended cells is not the upward rise of the bubble, but rather its rapid burst at the air-liquid interface (Chalmers 1994).

Consequently, the innovative bioreactor may also exert a protective effect on CHO cells through its distinct oxygenation strategy based on dynamic liquid–gas interfaces in the headspace.This approach removes the need for direct gas sparging in the liquid phase, thereby reducing bubble formation and mitigating associated cellular damage.

The results of the present study demonstrate that the innovative bioreactor significantly outperforms a traditional stirred-tank system in CHO cell fed-batch culture, particularly in terms of viable cell density (VCD), cell viability, and monoclonal antibody (mAb) titer.

The innovative bioreactor reached a peak VCD of 2.55 × 10⁷ cells mL^− 1^ on day 8 and maintained high viability (~ 91.7%) through day 10, with a final mAb titer of 1.3 ± 0.09 g L^− 1^. This represents a nearly two-fold increase over the titer achieved in the traditional system under similar process conditions (0.71 ± 0.006 g L^− 1^). It is important to note that cultures in the innovative bioreactor did not reach a clear production plateau by day 10. Therefore, the comparison reflects performance at this specific operational endpoint, which was determined by reaching the final working volume as a result of the feeding strategy, and may thus underestimate the full production potential of the system.

The significantly improved mAb productivity observed in the innovative bioreactor may be primarily attributable to the higher number of viable cells and their prolonged survival, rather than to an increase in cell-specific productivity. In particular, based on cell counts performed after trypsin treatment of samples recovered from the innovative bioreactor, it can be reasonably assumed that the actual cell number after day 8 may have been substantially higher than that estimated using conventional counting methods. The use of a slow-rotating drum rather than high-speed impellers likely contributed to this improved viability (Senger and Karim 2003; Li et al. 2020).

These results are in line with intensified fed-batch strategies, where high inoculation density (HID) and optimized feeding enable rapid biomass accumulation and increased productivity (Olin et al. 2024); Huang et al. 2010).

For example, Olin et al. (2023) demonstrated that combining N-1 perfusion and HID fed-batch culture improved productivity by up to 85%, even under standard feeding regimes. Similarly, a recent paper emphasizes that fed-batch processes with well-controlled nutrient supply and reduced stress conditions frequently reach titer of 2 g L^− 1^ in 10–14 days (Gibbons et al. 2023).

Peak viable cell density in the range of 2–3 × 10^7^cells mL^− 1^ could be usually achieved in a typical fed-batch process thanks to the advancements in cell line, media and process optimization (Xu et al. 2016; Bielser et al. 2018). In particular, perfusion cultures have been incrementally improved in recent years, and a typical bioreactor run could be achieved a stable cell concentration of 100 × 10^6^ cells mL^− 1^ (Tregidgo et al. 2023).

Maintaining high viability is critical for prolonged productivity and product quality. The innovative system showed > 91% viability at day 10, while the traditional system dropped to ~ 85%. High viability late in the culture process correlates with reduced proteolytic degradation, minimized host cell proteins (HCPs) release, and improved product consistency (Tait et al. 2012; Sahoo et al. 2024). HCPs are a complex group of impurities composed of thousands of different proteins produced by the host organism during the production of biologics and which may co-purify with the product of interest through downstream processing (Pilely et al. 2022). Trace amounts are seen in the final drug substance even after purification, with potential risk for clinic applications (Bracewell et al. 2015).

Lactate levels peaked in the first 3 days of culture and subsequently decreased in both bioreactor types, suggesting a shift from glycolytic overflow metabolism to lactate consumption. However, the process with the innovative bioreactor registered a lower lactate concentration of 1.35 g L^− 1^ at 10 days.

This observation is further supported by the lower apparent glucose-to-lactate yield measured in the innovative system (0.106 g/g), approximately half that of the stirred‑tank bioreactor (0.216 g/g) under comparable glucose supply. These values are consistent with literature reports for CHO fed‑batch processes, where glucose-to-lactate yields typically fall within the range of 0.1 to 0.3 g/g (Toussaint et al. 2016.

This metabolic shift has been widely reported in optimized CHO cultures and is associated with improved culture longevity and viability (Brunner et al. 2018). Lactate metabolism represents a critical aspect of industrial mammalian cell culture systems. Due to its detrimental effects on cellular performance and bioproduction efficiency, the mitigation of lactate accumulation is a central objective in cell line engineering and bioprocess optimization (Buchsteiner et al. 2018).

The ability to manage lactate accumulation is enhanced by modulating oxygen availability, as demonstrated in recent study by (Yin et al. 2025), where the authors demonstrated that DO has a dominant impact on lactate metabolism in the late stage of the process. Within this context, the lower apparent yield observed in the innovative bioreactor could indicate more efficient carbon utilization and a more favourable metabolic profile.

Overall, it is important to emphasize that the comparison performed in this study reflects differences at the system level, as the two bioreactors vary simultaneously in working volume, oxygen delivery mode, and mixing mechanism. Consequently, the observed improvements in cell growth, viability, and mAb production should be interpreted as the combined effect of the overall operational configuration rather than the isolated influence of individual engineering parameters.

We acknowledge that these results are based on laboratory‑scale experiments and that direct scale‑up is not straightforward. Although the innovative bioreactor showed encouraging performance, its potential industrial applicability cannot be fully assessed at this stage, as key product‑quality attributes (e.g., glycosylation, aggregation, fragmentation) were not evaluated in the present study. Therefore, any consideration of industrial relevance should be regarded as preliminary until comprehensive product‑quality analyses are performed.

Further studies are required to validate performance and to address scale‑up challenges. The implementation of feedback‑controlled feeding strategies (e.g., based on glucose, lactate, or Raman monitoring) may further improve culture stability and process productivity. Finally, the formation of cell aggregates (clumps) observed in the later stages of cultivation represents a critical aspect that should be carefully considered. Aggregation became significant after day 8, leading to an underestimation of viable cell density when measured without trypsin treatment. Moreover, clumps may create local gradients of nutrients and oxygen, potentially affecting cellular behavior and overall process performance. Accordingly, aggregate formation should be closely monitored and, where possible, mitigated, and will be the focus of future studies aimed at identifying strategies to limit aggregation.

## Conclusions

Suspension culture represents the most widely used platform for the production of biopharmaceuticals using CHO cells. In this context, we evaluated an innovative bioreactor developed by Eco-Sistemi srl, which may represent a promising alternative to conventional systems, supporting mammalian cell growth while maintaining high viability and enhancing mAb production.

However, further studies are required to better understand the system’s performance limits and to optimize operating conditions. It is also important to emphasize that, in mammalian cell bioprocessing, productivity alone is not sufficient to assess process performance. Critical quality attributes, such as glycosylation profile, aggregation, fragmentation, and host cell protein (HCP) contamination, must be carefully considered and could be the subject of future studies, together with scale-up investigations, to evaluate their behavior at larger scales.

## Supplementary Information

Below is the link to the electronic supplementary material.

Supplementary Material 1

## Data Availability

All data supporting the findings of this study are available within the paper.
