# Supplementary material for: Evaluation of a prototype bioreactor based on slowly rotating drum
Source: Cytotechnology. 2026 Jul 17;78(4):162. doi: 10.1007/s10616-026-01036-1 (PMC13379560; doi:10.1007/s10616-026-01036-1)
Supplement: Supplementary file 1 — Supplementary Material 1 [file 10616_2026_1036_MOESM1_ESM.docx]

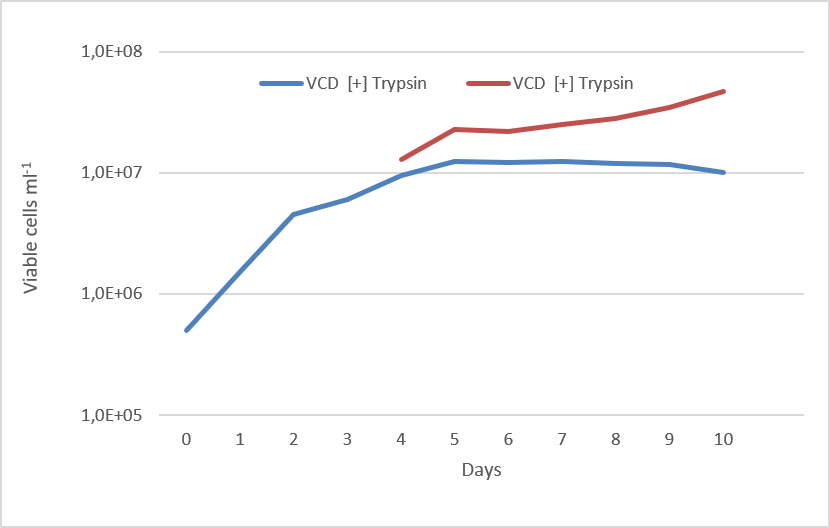


Figure S1. Comparison of viable cell density (VCD) over time measured with and without enzymatic dissociation (trypsin treatment). The samples from innovative bioreactor, which presented clump formation, were treated with a 0.5% trypsin-EDTA solution at 37°C for 8 minutes. The analysis highlights a progressive divergence between the two methods at later culture stages, with trypsin-treated samples showing higher VCD values, indicating a potential underestimation of cell concentration when cell aggregates are not dissociated.

Table S1. Estimated Gompertz model parameters for CHO growth on different bioreactor. The data were fitted to the model $y(t)=A\cdot exp\{-exp[(\mu\cdot e/A)(\lambda-t)+1]$, where A represents the maximum growth asymptote, μ the maximum specific growth rate, and λ the lag phase duration. Values are reported along with 95% confidence intervals (CI). The goodness of fit is expressed by the coefficient of determination (R²).

|  | A | CI95 A | μ | CI95 μ | λ | CI95 λ | R² |
| --- | --- | --- | --- | --- | --- | --- | --- |
| Traditional bioreactor | 7.06e+06 | ±4.9e+05 | 2.04e+06 | ±6.76e+05 | 0.662 | ±0.60 | 0.8943 |
| Innovative bioreactor | 1.75e+07 | ±2.7e+06 | 6.90e+06 | ± 5.29+06 | 2.988 | ±1.023 | 0.7455 |

# *Table S2. Two-way repeated-measures ANOVA results for the effect of bioreactor type and time on mAb production*

| Source | Df | Sum of Squares | Mean Square | F value | p-value |
| --- | --- | --- | --- | --- | --- |
| Reactor | 1 | 0.329 | 0.329 | 5.29 | 0.047 * |
| Time | 9 | 9.247 | 1.027 | 165.2 | < 0.001 *** |
| Reactor × Time | 9 | 1.876 | 0.208 | 33.5 | < 0.001 *** |
| Residuals | 18 | 0.112 | 0.006 | — | — |

Notes: * p < 0.05, *** p < 0.001. Repeated-measures ANOVA with time as within-subject factor.

# *Table S2.1. Post-hoc pairwise comparisons (Bonferroni correction)*

| Time (days) | p-value | Bonferroni-adjusted p-value | Significance |
| --- | --- | --- | --- |
| 1 | 2.57e-02 | 0.257 | ns |
| 2 | 2.65e-04 | 2.65e-03 | ** |
| 3 | 5.54e-01 | 1.000 | ns |
| 4 | 1.71e-01 | 1.000 | ns |
| 5 | 4.82e-01 | 1.000 | ns |
| 6 | 5.79e-02 | 0.579 | ns |
| 7 | 8.05e-03 | 0.0805 | ns |
| 8 | 1.35e-04 | 1.35e-03 | ** |
| 9 | 3.54e-05 | 3.54e-04 | *** |
| 10 | 3.51e-04 | 3.51e-03 | ** |

Significance: * p < 0.05; ** p < 0.01; *** p < 0.001; ns = not significant.
